# Inversion of nitrogen content in apple leaves using an explainable PSO-CNN model

**DOI:** 10.3389/fpls.2026.1789139

**Published:** 2026-04-21

**Authors:** Yuyang Ma, Xicun Zhu, Meixuan Li, Cheng Li, Dong Lv

**Affiliations:** 1 College of Resources and Environment, Shandong Agricultural University, Tai’an, China; 2 National Engineering Research Center for Efficient Utilization of Soil and Fertilizer Resources, Tai’an, China

**Keywords:** apple tree leaves, hyperspectral, inversion, nitrogen content, phenological period, PSO-CNN, SHAP

## Abstract

**Introduction:**

The integration of hyperspectral technology with machine learning and deep learning algorithms offers an effective method for accurately and non-destructively estimating the percentage of nitrogen in apple tree leaves, as well as for rapid nutrient diagnosis.

**Methods:**

This study was conducted in apple orchards in Qixia City, Shandong Province, where hyperspectral data were collected from Red Fuji apple trees during the new-shoot-stop-growing stage (NSS) and the autumn-shoot-stop-growing stage (ASS). Following hyperspectral preprocessing and characteristic wavelength selection, regression models—including random forest (RF), support vector machine (SVM), convolutional neural network (CNN), and particle swarm optimization convolutional neural network (PSO-CNN)—were developed and compared.

**Results:**

The results showed that CNN significantly outperformed RF and SVM, and that PSO-CNN further improved prediction performance. The PSO-CNN model achieved an R² of 0.886 on the training set and an R² of 0.774, an RMSE (%) of 0.095, and an *RPD* of 2.086 on the test set. Validation using samples from different phenological stages demonstrated acceptable prediction accuracy (*R^2^* = 0.625, *RMSE* = 0.173, *RPD* = 1.529), with uniformly distributed errors and no systematic bias, indicating good generalization capability and stability. SHAP analysis revealed that the PSO-CNN model primarily relied on the near-infrared and short-wave infrared bands, where reflectance was negatively correlated with nitrogen content.

**Discussion:**

These spectral regions are closely associated with leaf biochemical structure, suggesting that the model predicts nitrogen content by capturing spectral information related to leaf biochemical characteristics. Overall, the PSO-CNN model improves nitrogen prediction performance by expanding the hyperparameter search space while preserving the CNN architecture, enabling rapid and accurate nutrient diagnosis in apple leaves.

## 1 Introduction

Nitrogen is a fundamental nutrient regulating plant growth and development and plays a critical role in determining both yield and fruit quality in apple production (Ye et al., 2020). Apple is one of the most economically important fruit crops in China, accounting for over half of the global cultivation area and production, and contributing substantially to rural economies (Chen et al., 2024). Among the major production regions, Shandong Province accounts for approximately one-quarter of the national apple output (Shi et al., 2024). Accurate estimation of leaf nitrogen content is essential for assessing the nutritional status of apple trees and guiding fertilization strategies. Conventional methods, such as chemical analysis, are destructive, labor-intensive, and time-consuming, thereby limiting their applicability to real-time monitoring (Marang et al., 2021). In contrast, hyperspectral techniques provide a rapid and non-destructive alternative and have been widely adopted for crop nutrient assessment.

Hyperspectral techniques have been extensively utilized to assess nutrient status in fruit crops. Both imaging and non-imaging hyperspectral data, combined with linear and nonlinear models, have been employed to estimate canopy nitrogen content in pear orchards (Sun et al., 2025) and to diagnose nitrogen status in winter rape and summer maize (Tang et al., 2025b). Early studies primarily used linear regression models. For instance, Zhu et al. (2010) accurately predicted nitrogen content in apple blossoms under small-sample conditions. However, these approaches are often affected by multicollinearity and autocorrelation. To overcome these limitations, Gao et al. (2017) applied partial least squares regression to estimate leaf nitrogen content during the growth stage of new apple shoots. However, its sensitivity to outliers and limited capacity to model nonlinear relationships constrained its performance (Gao et al., 2017).

Machine learning methods have been introduced to enhance predictive capabilities. Support vector machine regression (SVMR) has demonstrated strong robustness to noise and good generalization performance on small datasets (Li et al., 2018). Artificial neural networks, including backpropagation neural networks (BPNNs), can capture complex nonlinear relationships through adaptive weight optimization (Yang et al., 2018). By integrating spectral indices and red-edge parameters, BPNN-based models have accurately reconstructed canopy nitrogen content in apple orchards (Li et al., 2022). Additionally, ensemble-based approaches, such as optimized extreme learning machine models, have shown improved performance in nutrient estimation (Chen et al., 2022).

Despite these advances, individual machine learning models often exhibit limited adaptability under complex environmental conditions, with constrained generalization ability and prediction accuracy. Ensemble learning methods enhance model robustness by integrating multiple base learners and optimizing data representation, therefore mitigating overfitting and underfitting (Feng et al., 2020). For example, hybrid RF–SVM models have achieved higher accuracy in predicting apple canopy chlorophyll content compared to single models (Bai et al., 2021), while XGBoost has demonstrated superior performance in estimating nitrogen indices in summer maize (Tang et al., 2025a).

However, ensemble learning is contingent on its base learners and lacks a unified feature-learning framework, which limits its ability to capture complex nonlinear relationships efficiently. When processing high-dimensional hyperspectral data, a large number of base learners are often required, leading to increased computational costs. Deep learning models, particularly convolutional neural networks (CNNs), offer an effective alternative by integrating feature extraction and representation learning within a unified architecture (Wang et al., 2022). CNNs have been successfully applied in agricultural contexts, including nitrogen deficiency detection in rice (Sethy et al., 2020) and yield prediction using UAV-derived data (Nevavuori et al., 2019).

Nevertheless, conventional CNNs are prone to converging to local optima. Hyperparameter tuning typically relies on empirical trial-and-error or grid search methods, which are computationally expensive. The particle swarm optimization (PSO) algorithm offers an efficient global optimization strategy by enabling information exchange among particles to explore the search space (Piotrowski et al., 2020). By dynamically updating particle positions and velocities, PSO can guide CNN training away from local optima and enhance optimization efficiency. PSO-CNN frameworks have demonstrated a favorable balance between accuracy and computational cost in image classification tasks (Fernandes Junior and Yen, 2019). Concurrently, model interpretability has become increasingly important. Shapley additive explanations (SHAP), derived from cooperative game theory, have been widely used to quantify feature contributions and interpret deep learning models (Zhong et al., 2024).

Despite these advancements, the application of PSO-CNN models for hyperspectral inversion of nitrogen content in apple leaves remains largely unexplored. Furthermore, the relationship between spectral features and nitrogen prediction has not been thoroughly interpreted. Therefore, this study evaluates the performance of a PSO-CNN model for estimating apple leaf nitrogen content and compares it with conventional CNN approaches. Additionally, SHAP analysis is employed to elucidate the contribution of characteristic spectral bands across different phenological stages, providing insights into the underlying mechanisms of nitrogen estimation and supporting more efficient nutrient management in orchards.

## 2 Materials and methods

### 2.1 Research area

**Figure 1:**
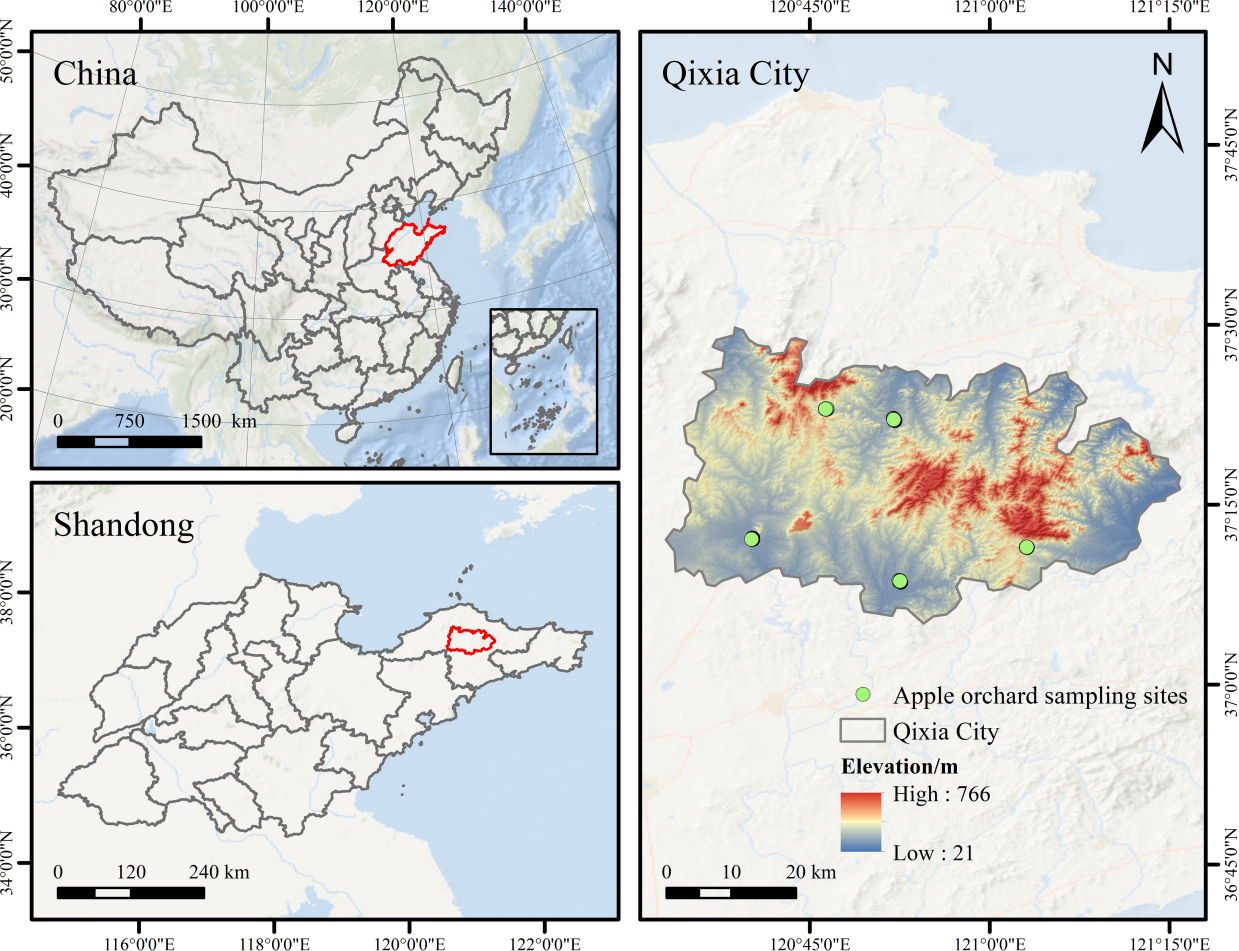
Location of the research area.

**Figure 2:**
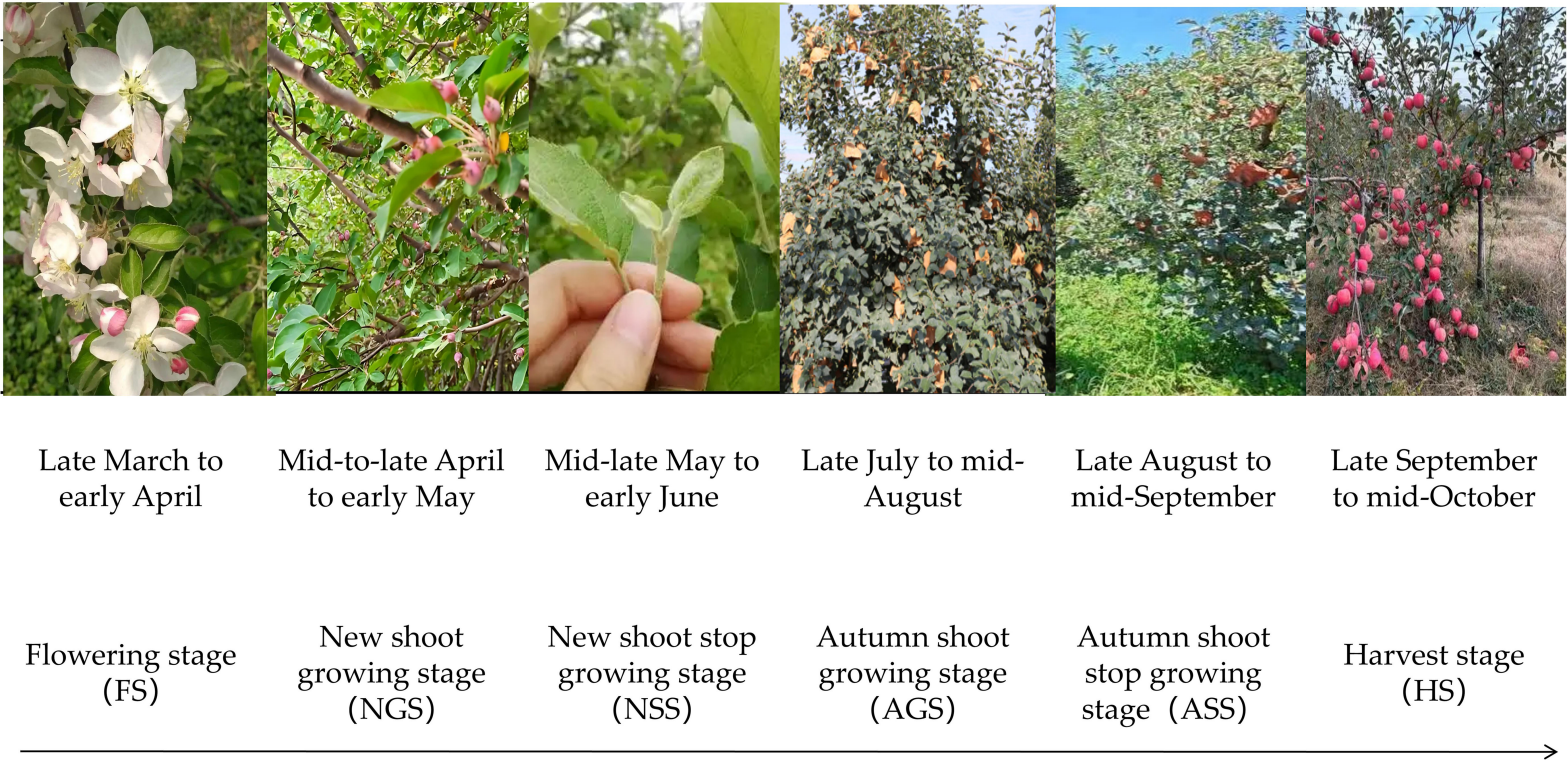
Diagram of phenological periods of apple trees.

The study was conducted in Qixia City, Shandong Province, China (120°33′–121°15′E, 37°05′–37°32′N), located in the inland region of the Jiaodong Peninsula (**Figure 1**). The area covers approximately 1,793 km² and features a hilly terrain and subtropical monsoon climate. The mean annual temperature is 11.4 °C, with a frost-free period of about 209 days and annual precipitation ranging from 640 to 846 mm. **Figure 2** illustrates the main phenological stages of apple tree growth. Based on local orchard management practices, fertilization is typically applied during the new shoot stop (NSS) and the autumn shoot stop (ASS) stages.

### 2.2 Apple leaf sampling and data acquisition

Three apple orchards in Qixia City were sampled in early June 2023 (NSS) for model development, while two additional orchards were sampled in early September 2024 (ASS) for validation. Sampling points were evenly distributed across all five orchards (**Figure 1**). Fuji apple trees of similar age were selected, and three healthy, intact leaves were collected from each tree at the east, west, south, and north canopy positions. Samples were immediately sealed in plastic bags, stored with ice packs, labeled, and transported under refrigerated conditions. In total, 132 samples were collected in June 2023 and 120 samples in September 2024.

#### 2.2.1 Spectral acquisition

Spectral data were acquired utilizing an ASD FieldSpec 4 spectrometer (350–2,500 nm). The 350–1,000 nm range was sampled at 1.4 nm intervals with a 3 nm resolution, and the 1,000–2,500 nm range at 1.1 nm intervals with a 6 nm resolution. Measurements were conducted in a darkroom under controlled illumination. Leaf surfaces were gently cleaned before measurements. Each leaf was placed flat on a black background with near-zero reflectance. The spectrometer (25°field of view) was positioned vertically at a height of 0.10 m above the sample. A 50 W halogen lamp, placed 0.50 m from the sample at an angle of 60°, served as the light source. For each sample, 10 spectra were recorded and averaged. White reference calibration was performed periodically to maintain stability. The 350–400 nm range was excluded owing to noise and illumination sensitivity.

#### 2.2.2 Nitrogen content determination

Leaves employed for spectral measurements were oven-treated at 80 °C for 15–30 min and then dried at 60 °C to constant weight. The dried samples were ground into powder, digested with H_2_SO_4_–H_2_O_2_, and analyzed for nitrogen content utilizing the Kjeldahl method. Outliers were removed utilizing the Mahalanobis distance method, yielding 125 valid samples for the NSS period (2023) and 118 samples for the ASS period (2024). The NSS dataset (n = 125) was adopted for model development, while the ASS dataset (n = 118) served as an independent validation set. The SPXY (Sample set partitioning based on joint X–Y distance) algorithm was applied to partition the NSS dataset into training (n = 75) and test (n = 50) sets, ensuring representative distribution in both spectral (X) and response (Y) spaces (Li et al., 2020). **Table 1** presents descriptive statistics of the sample sets, indicating relatively low variability within the dataset.

**Table 1:** Nitrogen content (%) samples statistics for apple trees leaves (ASS, Autumn shoot stop growing stage; NSS, new shoot stop growing stage).

Dataset	Sample number (n)	Maximum value (%)	Minimum value (%)	Mean value (%)	Standard deviation	Coefficient of variation (%)
Total (ASS)	118	3.752	2.235	2.795	0.248	8.873
Total (NSS)	125	2.873	2.035	2.479	0.201	8.108
Training set (NSS)	75	2.851	2.036	2.489	0.203	8.156
Test set (NSS)	50	2.873	2.035	2.465	0.199	8.073

### 2.3 Spectral preprocessing

Raw spectral data were preprocessed using Savitzky–Golay smoothing (SG), second derivative (SD), multiplicative scatter correction (MSC), and standard normal variate (SNV).

SG smoothing reduced noise while preserving spectral features (Du et al., 2011) (Equation 1). SD transformation minimized baseline drift and enhanced spectral features such as peaks and inflection points (Czarnecki, 2015). MSC corrected scattering effects and improved spectral consistency across samples (Dhanoa et al., 2023) (Equation 2). SNV normalized spectral data by removing scaling effects and variations caused by particle size and light scattering (Bi et al., 2016) (Equation 3).

(1) $${\text{X}}_{\text{k},\text{smooth}}=\bar{{\text{x}}_{\text{k}}}=\frac{1}{\text{H}}\sum_{\text{i}=-\text{w}}^{+\text{w}}{\text{x}}_{\text{k}+\text{i}}{\text{h}}_{\text{i}}$$

(2) $$\begin{array}{c} {\text{MSC}}_{\text{i}}=\frac{{\text{R}}_{\text{i}}-{\text{b}}_{\text{i}}}{{\text{k}}_{\text{l}}} \end{array}$$

(3) $$\begin{array}{c} {\text{X}}_{\text{SNV}}=\frac{\text{x}-\bar{\text{x}}}{\sqrt{\frac{\sum_{\text{k}=1}^{\text{m}}({\text{x}}_{\text{k}}-{\bar{\text{x})}}^{2}}{\text{m}-1}}} \end{array}$$

In Equation 1, k denotes the spectral band point, w represents the moving window width, and $\bar{{\text{x}}_{\text{k}}}$ is the average value of W wavelength points surrounding the target wavelengths, K and K point. ${\text{x}}_{\text{k}+\text{i}}$ denotes the original spectral reflectance at position k+i. $\frac{{\text{h}}_{\text{i}}}{\text{H}}$ is the smoothing coefficient, which is obtained by the least squares principle. H is the normalization factor defined as $H=\sum_{\text{i}=-\text{w}}^{+\text{w}}{\text{h}}_{\text{i}}$, where h_i_ is the Savitzky–Golay smoothing coefficient corresponding to the *i*-th point in the moving window. In Equation 2, R_i_ represents the reflectivity of the original spectrum at the *i*-th line, while b_i_ and k_1_ denote the baseline translation and offset, respectively. In Equation 3, x, $\bar{\text{x}}$, and x_k_denote the original reflectance value of a spectral variable, mean reflectance, and reflectance value of the *k*-th spectral variable, respectively. The variable m denotes the total number of spectral variables, with k=1,2,3…m.

### 2.4 Feature wavelength screening

Feature wavelengths were selected utilizing a hybrid approach that combines competitive adaptive reweighted sampling (CARS) and successive projections algorithm (SPA). CARS was first applied to remove uninformative variables and retain wavelengths with high predictive relevance (Li et al., 2009). However, CARS can still retain redundant variables. To address this, SPA was subsequently employed to select characteristic wavelengths with minimal collinearity via a forward selection procedure (Soares et al., 2013). This combined strategy reduced variable redundancy and dimensionality, thereby improving model stability and generalization performance (Tang et al., 2014).

### 2.5 Machine learning regression models

Random forest (RF) and support vector machine (SVM) were selected as comparative models due to their proven performance in hyperspectral inversion of vegetation biochemical parameters, particularly leaf nitrogen content (Zha et al., 2020).

RF was utilized to capture nonlinear relationships between spectral features and nitrogen content. The model utilized reflectance at 15 selected wavelengths as input and leaf nitrogen content as the output. It was configured with 300 trees, a maximum depth of 8, and a minimum of 3 samples per leaf node to improve model stability and reduce overfitting (Li et al., 2024).

An SVM with a radial basis function (RBF) kernel was applied to model nonlinear relationships under limited sample conditions (Elen et al., 2022). The penalty parameter (C) and kernel parameter (γ) were optimized via grid search on the training set, with search ranges set to 2^−5 – 2^9 for C and 2^−15 – 2^1 for γ, ensuring a balance between model complexity and generalization performance.

### 2.6 Deep learning regression models

Deep learning models were applied to capture nonlinear relationships in hyperspectral data (Peng et al., 2020; Wang et al., 2021). Following SG–SD preprocessing and CARS–SPA feature selection, the selected wavelengths were utilized as input variables, with leaf nitrogen content as the output, to construct CNN and PSO-CNN models.

#### 2.6.1 Convolutional neural network model

A CNN was employed to model the relationship between spectral features and leaf nitrogen content, leveraging its strong feature extraction and generalization capabilities for crop nutrient prediction (Liao et al., 2024). The network comprised an input layer, convolutional layers, pooling layers, fully connected layers, and an output layer. It was trained utilizing forward propagation and backpropagation algorithms (Fukushima, 1980; Tran et al., 2019; Chew et al., 2020).

A one-dimensional CNN architecture was designed to balance model complexity and generalization under limited sample conditions (Li et al., 2020). The detailed architecture and hyperparameter settings are provided in **Table 2**. The network included two convolutional layers, two activation layers, two pooling layers, one dropout layer, one fully connected layer, and an output layer. ReLU activation functions were used to introduce nonlinearity (Munender and Pravendra, 2021), and max-pooling layers were applied to reduce feature dimensionality (Li et al., 2025). To mitigate overfitting, a dropout layer (rate = 0.5) was inserted before the fully connected layer (Gavahi et al., 2023), and L2 regularization (λ = 0.001) was applied during training (Deng et al., 2025).

**Table 2:** The CNN architecture and training hyperparameters.

Parameter	Value
Convolution layers	2
Convolution kernels	8 per layer
Kernel size	3×1
Activation function	ReLU
Pooling type	Max pooling
Dropout rate	0.5
Regularization	L2 (λ = 0.001)
Batch size	32
Optimizer	Adam
Initial learning rate	0.001
Learning rate decay	0.1
Iterations	500

Each convolutional layer employed 8 kernels of size 3 × 1 to extract local spectral features (Hong and Chan, 2023). The model was trained utilizing the Adam optimizer with an initial learning rate of 0.001 and a decay factor of 0.1 (Zhou and Xiong, 2023). Training utilized a batch size of 32 and a maximum of 500 iterations, with early stopping based on validation loss to prevent overfitting (Jiang et al., 2025).

#### 2.6.2 Particle swarm optimization–convolutional neural network model

**Figure 3:**
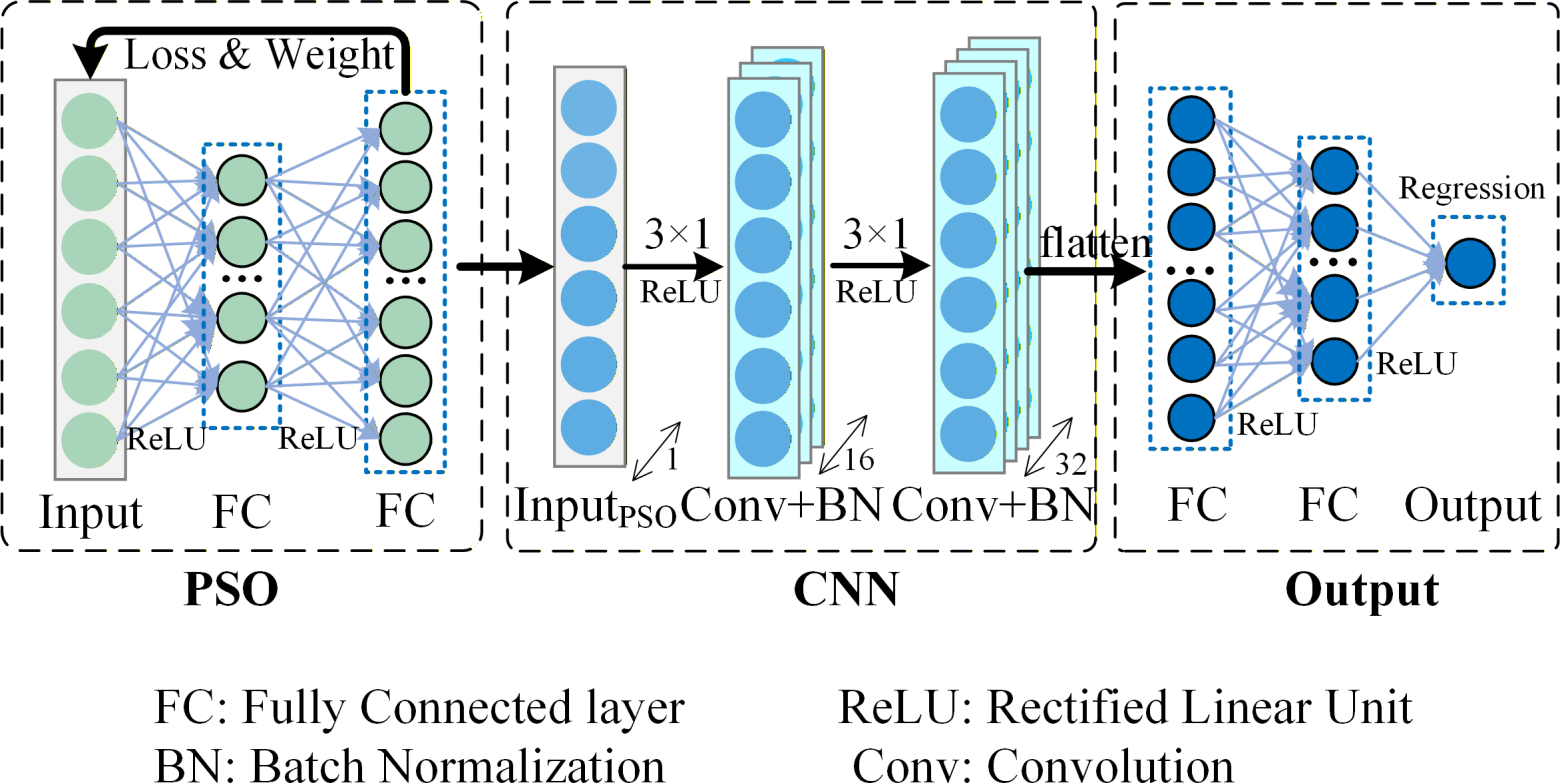
Process diagram of PSO optimization of CNN structure.

Particle swarm optimization (PSO) was employed to optimize the hyperparameters of the CNN model (Kennedy and Eberhart, 1995). PSO performs global optimization by iteratively updating particle positions and velocities within the search space (Kameyama, 2009; Kingma and Ba, 2014). Here, PSO was utilized to optimize three key CNN hyperparameters: learning rate, batch size, and regularization coefficient. **Table 3** presents the corresponding search ranges of these parameters. **Figure 3** illustrates the PSO-CNN model architecture.

**Table 3:** Search ranges of the CNN hyperparameters optimized by PSO.

Hyperparameter	Search range
Learning rate	0.0005–0.01
Batch size	16–512
Regularization coefficient	0.0001–0.1

The learning rate was searched within the range of 0.0005–0.01 to ensure stable convergence (Hariri and Avşar, 2022). The batch size was varied from 16 to 512 to balance computational efficiency and memory constraints (Yuan et al., 2024). The regularization coefficient was optimized to control model complexity and reduce overfitting. The particle population size was set to 20, and the maximum number of iterations was 15, following recommendations for small-sample optimization problems (Piotrowski et al., 2020). Similar settings have been reported in PSO-based deep learning studies (Hu and Gu, 2025). To ensure comparability, the CNN architecture, including the number of layers and convolution kernel configuration (8 kernels with a size of 3 × 1), was kept consistent with the baseline model. The fitness function was defined as the root mean square error (*RMSE*) between predicted and observed leaf nitrogen content on the test set.

### 2.7 Model accuracy evaluation

Model performance was evaluated utilizing the coefficient of determination (*R²*), root mean square error (*RMSE*), and relative percent difference (*RPD*) (Deng et al., 2025). Higher *R²* and lower *RMSE* values indicate better model performance. *RPD* was employed to assess predictive reliability, with values < 1.4 indicating poor performance, 1.4–2.0 indicating moderate predictive capability, and > 2.0 indicating strong predictive capability. The corresponding formulas are as follows:

(4) $$\begin{array}{c} R^{2}=1-\frac{\sum_{\text{i}=1}^{\text{n}}{\left({\text{y}}_{\text{i}}-\hat{{\text{y}}_{\text{i}}}\right)}^{2}}{\sum_{\text{i}=1}^{\text{n}}{\left({\text{y}}_{\text{i}}-\bar{{\text{y}}_{\text{i}}}\right)}^{2}} \end{array}$$

(5) $$\begin{array}{c} RMSE=\sqrt{\frac{\sum_{\text{i}=1}^{{\text{I}}_{\text{p}}}{\left({\text{y}}_{\text{i}}-\hat{{\text{y}}_{\text{i}}}\right)}^{2}}{{\text{I}}_{\text{p}}}} \end{array}$$

(6) $$\begin{array}{c} RPD=\frac{\sqrt{\frac{\sum_{\text{i}=1}^{\text{n}}{\left(\hat{{\text{y}}_{\text{i}}}-\bar{{\text{y}}_{\text{i}}}\right)}^{2}}{\text{n}-1}}}{RMSE} \end{array}$$

In Equations 4, 6, n, ${\text{y}}_{\text{i}}$, $\hat{{\text{y}}_{\text{i}}}$, and $\bar{{\text{y}}_{\text{i}}}$ denote the number of samples, actual value of the sample, predicted value, and actual mean value of the sample. In Equation 5, ${\text{I}}_{\text{p}}$ represents the number of predicted samples.

### 2.8 Model explainability analysis

Model interpretability was assessed utilizing the Shapley Additive Explanations (SHAP) method to quantify the contribution of input wavelength features to leaf nitrogen content prediction. SHAP estimates feature importance by computing each variable’s marginal contribution to the model output and provides both global and local interpretability (García and Aznarte, 2020). The Shapley value for each feature was calculated as its weighted contribution across all possible feature combinations, reflecting its influence on the prediction.

(7) $$\begin{array}{c} \varphi_{i}=\sum \{S\subseteq \{x_{-}i\}\}[w(S,i)*(f(S\cup \{i\})-f(S))] \end{array}$$

In Equation 7, $\varphi_{i}$ denotes the Shapley value of feature *i*, *S* represents all possible subsets of features excluding feature *i*, and w(S,i) is the weight assigned to the subset *S* combined with feature *i*. In addition, f(S∪{i}) and f(S) denote the model’s predicted values with and without feature *i*, respectively.

## 3 Results

### 3.1 Hyperspectral characteristics of apple leaves with different nitrogen contents

**Figure 4:**
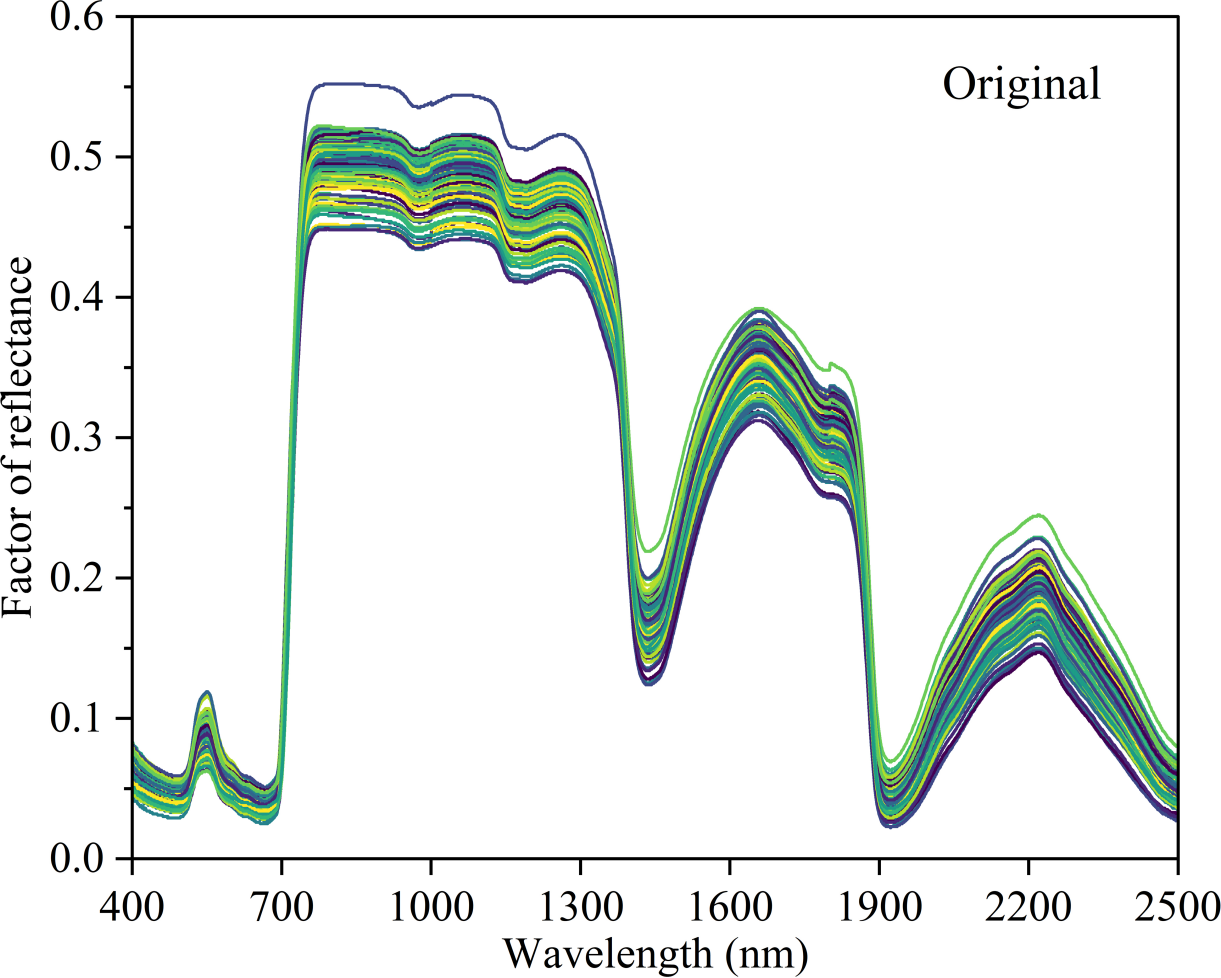
Original spectral reflectance curve (different colored curves represent different samples). The sample size was n=125.

Hyperspectral reflectance data from 125 leaf samples with varying nitrogen content are presented in **Figure 4**. Although the magnitude of reflectance differed among samples, the overall spectral patterns remained consistent. In the visible region (400–700 nm), reflectance was low with limited variability. A sharp increase occurred near 700 nm, corresponding to the red-edge region. In the near-infrared region (780–1,300 nm), reflectance was relatively high and exhibited greater variability among samples. In the short-wave infrared region, distinct absorption features appeared near 1,400 nm and 1,900 nm, where reflectance decreased markedly. Beyond these regions, reflectance increased again, forming local maxima around 1,700 nm and 2,200 nm, accompanied by enhanced variability among samples.

**Figure 5:**
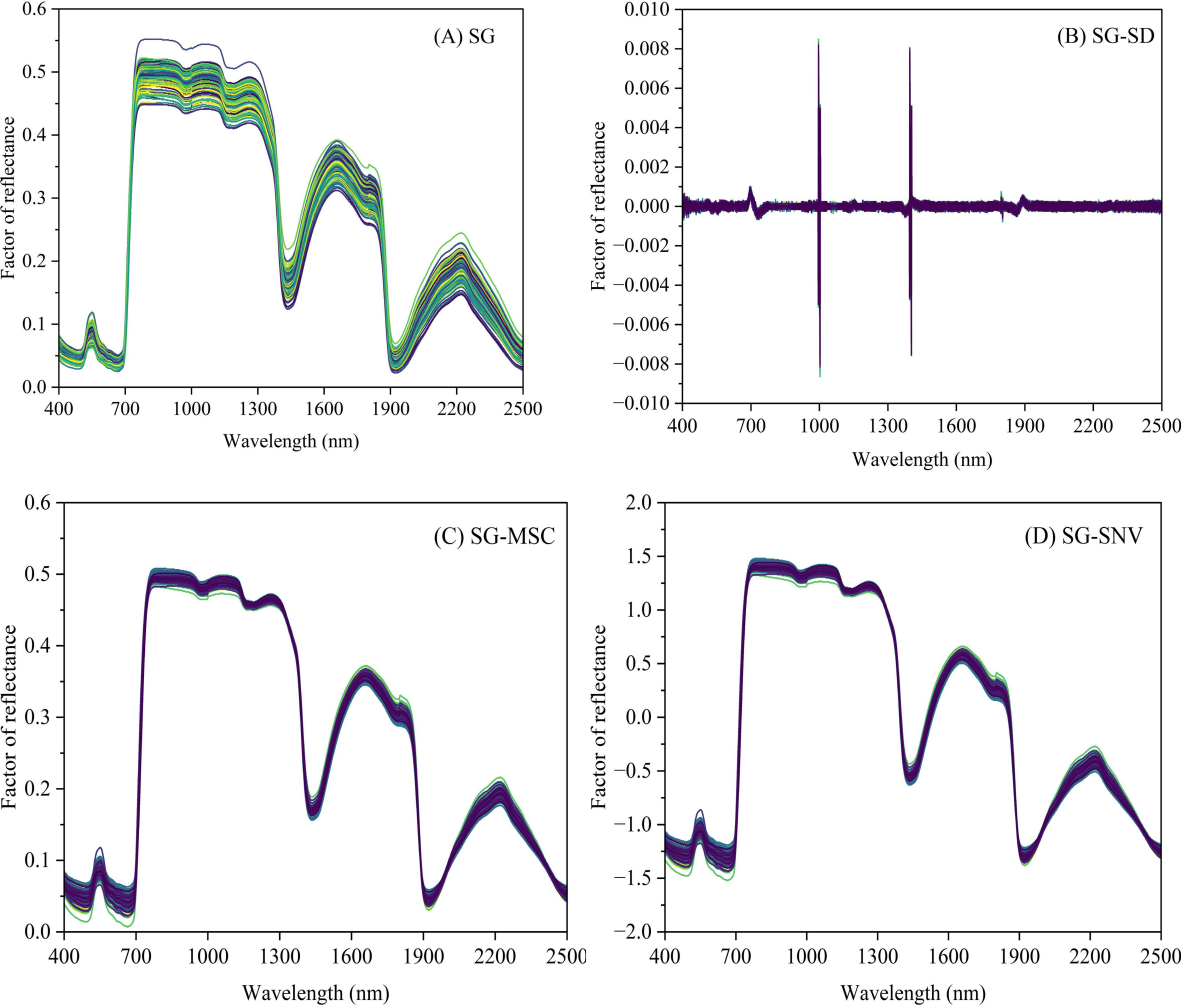
Leaves spectral curves: **(A)** after SG; **(B)** after SG-SD; **(C)** after SG-MSC; **(D)** after SG-SNV (Abbreviations are as follows: SG, Savitzky-Golay smoothing; SD, second derivative; MSC, multiplicative scatter correction; SNV, standard normal variation). The sample size processed by each of the above four preprocessing methods above was n=125.

The original spectra were smoothed using the SG method and subsequently processed using SD, MSC, and SNV transformations (**Figure 5**). The SG method effectively reduced spectral noise, while the SD transformation enhanced spectral variations, particularly at peaks, troughs, and inflection points. Both MSC and SNV exhibited trends similar to the smoothed spectra and reduced inter-sample variability; however, SNV further expanded the range of variation.

#### 3.1.1 Correlation analysis between spectral reflectivity and different nitrogen contents

**Figure 6:**
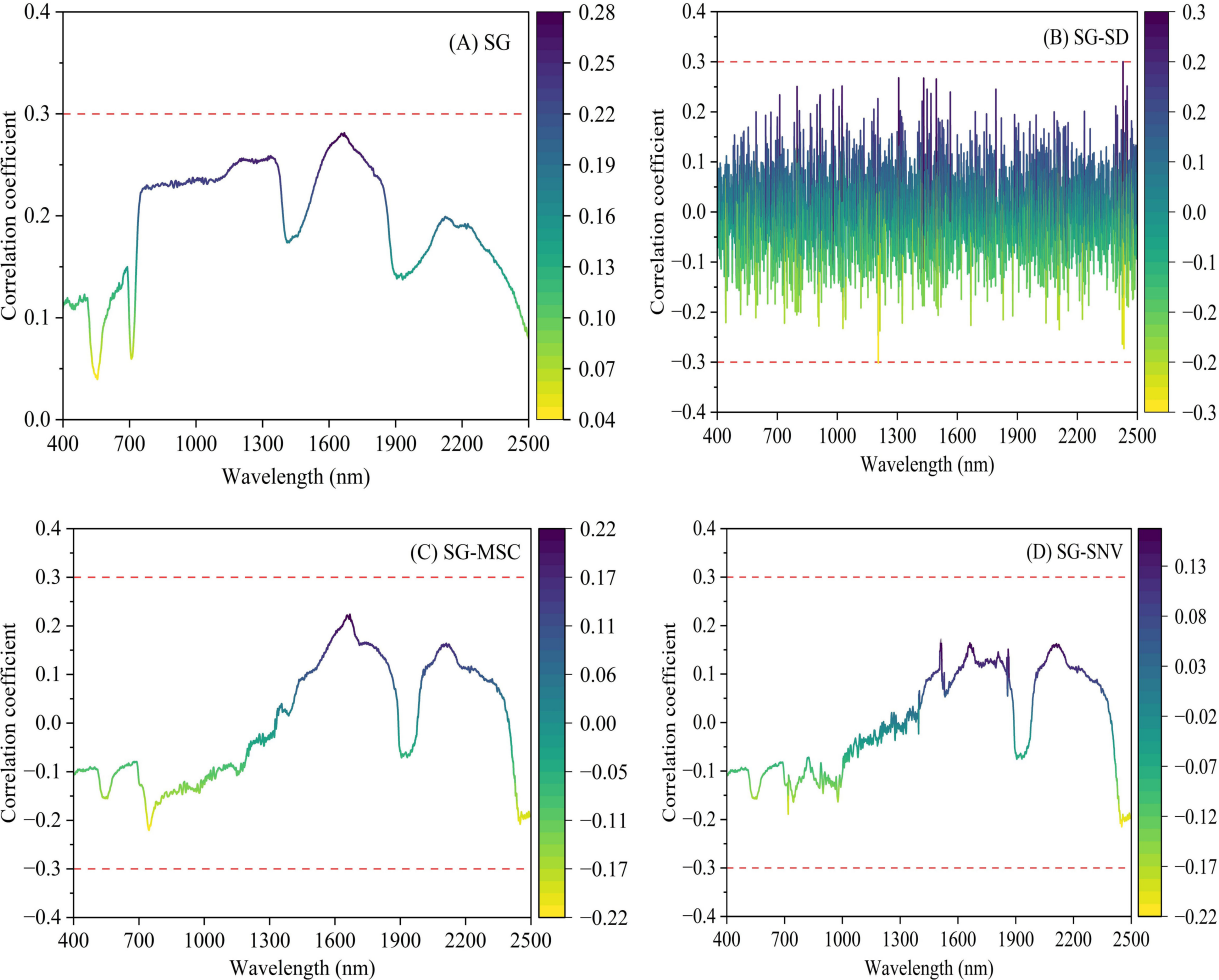
Correlation coefficient curve color mapping: **(A)** after SG; **(B)** after SG–SD; **(C)** after SG–MSC; **(D)** after SG–SNV. (Abbreviations are as follows: SG, Savitzky-Golay smoothing; SD, second derivative; MSC, multiplicative scatter correction; SNV, standard normal variation). The number of samples used to calculate the correlation coefficients between reflectance at each wavelength and nitrogen content was n=125.

Pearson correlation analysis was conducted to evaluate the relationship between spectral reflectance (after applying four preprocessing methods) and leaf nitrogen content. The results are presented in **Figure 6**. Following SG preprocessing, correlations remained weak, with maximum coefficients below 0.3. The SD transformation enhanced the correlation, with some wavelengths approaching values close to 0.3. In contrast, MSC and SNV transformations decreased the absolute correlation coefficients, which remained well below 0.3. Overall, the combination of SG and SD preprocessing yielded stronger correlations compared to the other methods.

### 3.2 CARS-SPA feature wavelength screening

Following SG smoothing and SD transformation, spectral reflectance and the corresponding nitrogen content were used to select features via the CARS-SPA approach. Initially, CARS selected 102 characteristic wavelengths based on the SG–SD spectra. To further reduce redundancy, SPA was applied to refine the feature set. SPA identified an optimal subset of 15 wavelengths, corresponding to the minimum root mean square error (*RMSE*) of 0.1540. The selected wavelengths were 411, 450, 522, 539, 917, 975, 1359, 1425, 2045, 2181, 2215, 2324, 2382, 2387, and 2394 nm.

### 3.3 Nitrogen Prediction of Apple Tree Leaves based on Machine Learning and Deep Learning Models

#### 3.3.1 Prediction of nitrogen content in apple leaves based on RF and SVM

**Figure 7:**
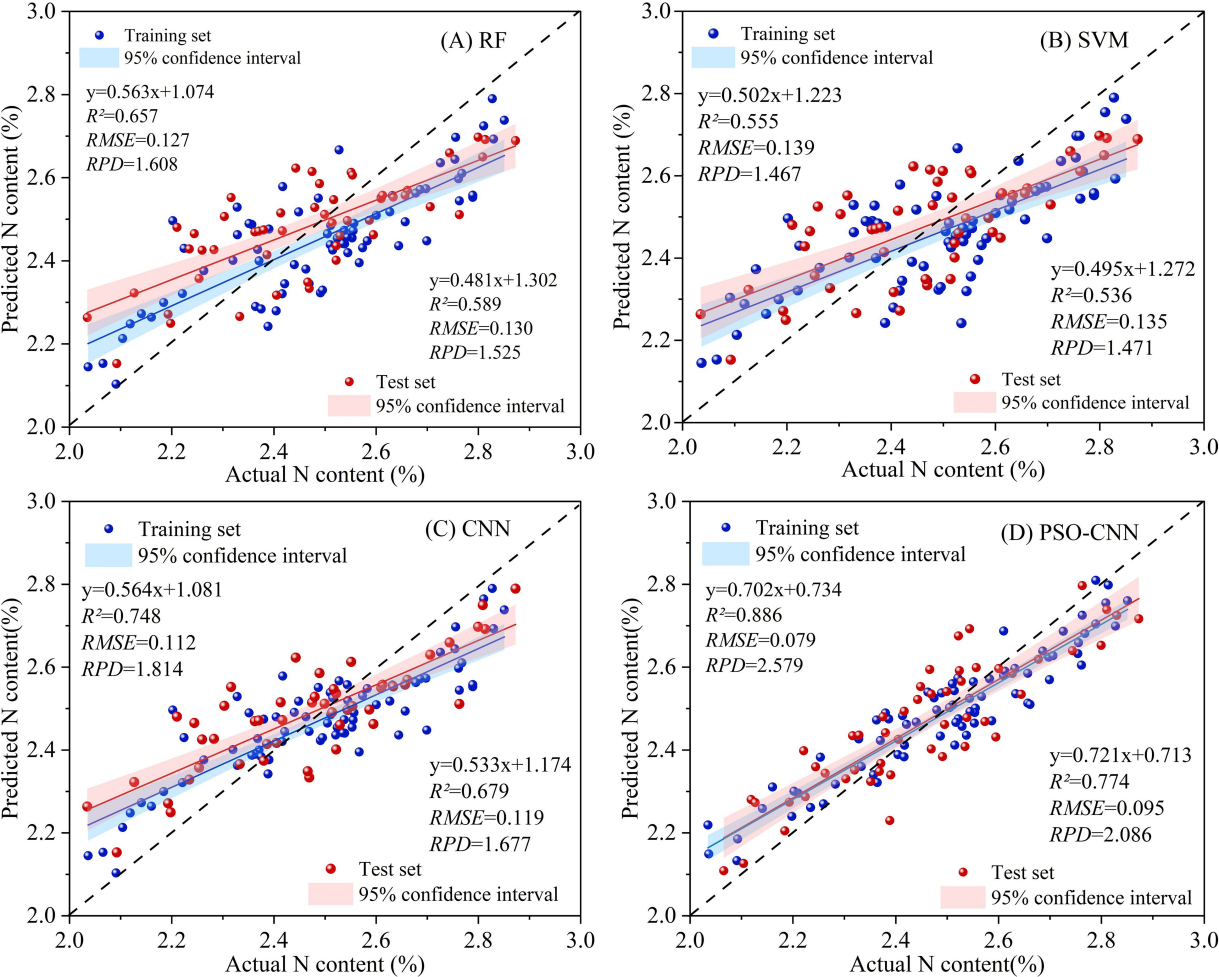
Scatter plot of model prediction results: **(A)** after RF; **(B)** after SVM; **(C)** after CNN; **(D)** after PSO-CNN. (Abbreviations are as follows: RF, random forest; SVM, support vector machine; CNN, convolutional neural network; PSO-CNN, particle swarm optimization convolutional neural network). In the four regression models mentioned above, the number of training set samples used is n=75, and the number of test set samples is n=50.

**Figure 7A, B** present the prediction results of the RF and SVM models, respectively. Overall, the RF model demonstrated superior predictive performance compared to the SVM model. Specifically, the RF model achieved an *R²* value exceeding 0.6 on the training set, with *RPD* values greater than 1.5 for both the training and test sets. In contrast, the SVM model exhibited lower performance, with an *R²* slightly above 0.5 and *RPD* values marginally exceeding 1.4. For both models, the fitted regression lines deviated from the ideal 1:1 line, with slopes around 0.5, indicating a systematic bias characterized by overestimation at low values and underestimation at high values. The 95% confidence intervals shown in **Figure 7A, B** further illustrate the uncertainty associated with the model predictions.

#### 3.3.2 Prediction of nitrogen content in apple leaves based on CNN

**Figure 8:**
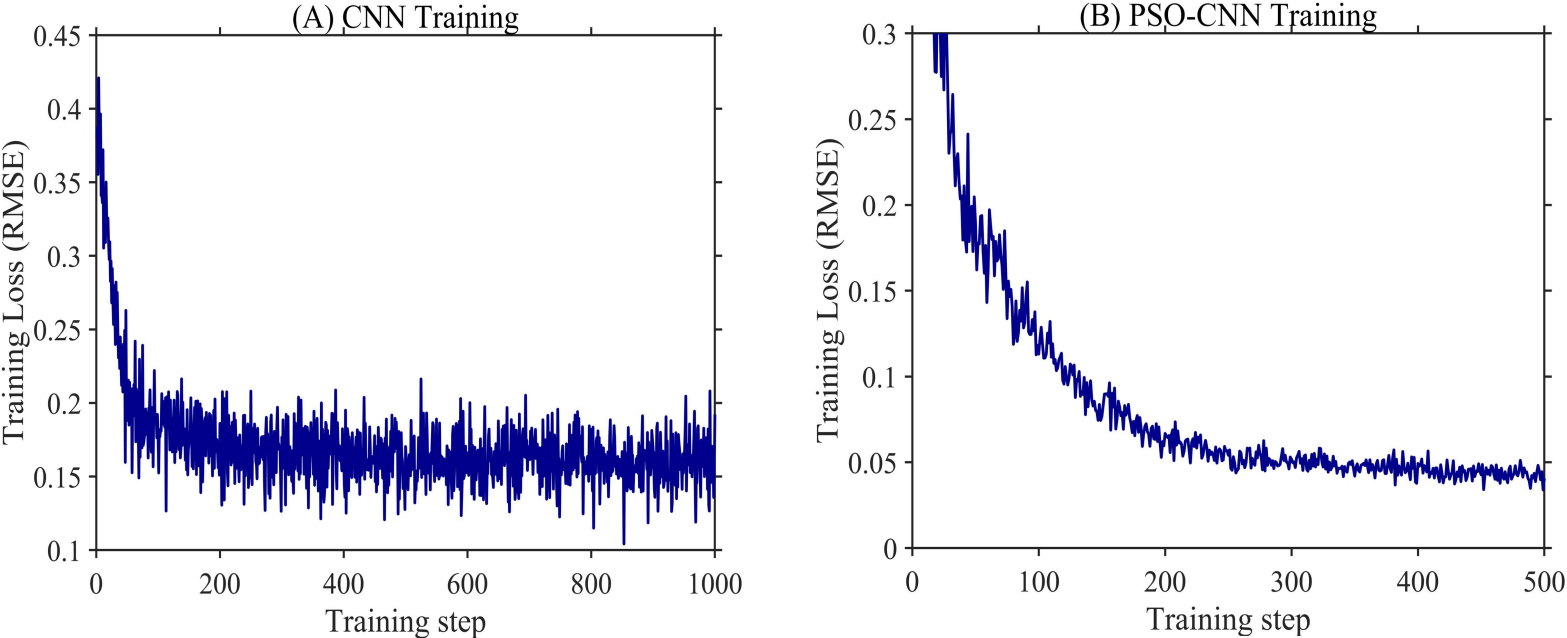
Training loss curves of **(A)** the CNN model and **(B)** the PSO-CNN model. The loss is measured by root mean square error (RMSE). The x-axis denotes the training steps, and the y-axis denotes the RMSE loss. Curves represent the mean values over n = 10 independent runs. The PSO-CNN model demonstrates faster convergence and achieves a lower final loss compared to the baseline CNN model.

**Figure 8A** illustrates the convergence behavior of the CNN model during training. The training *RMSE* decreased rapidly in the initial phase and then gradually stabilized, displaying a typical convergence pattern. After the initial decline, *RMSE* fluctuations diminished and remained stable at approximately 0.15 in the later stages, with no signs of divergence, indicating a stable training process and satisfactory convergence.

As shown in **Figure 7C**, the CNN model demonstrated improved predictive performance compared to the RF and SVM models. The training set achieved an *R²* > 0.7 and an *RPD* > 1.7, while the test set exhibited slightly lower but comparable performance, with an *R²* above 0.6, an *RMSE* of 0.119, and an *RPD* of 1.677. The scatter distribution in **Figure 7C** reveals that most predicted values are concentrated within the 2.4%–2.8% range, indicating a reduction in systematic bias, particularly the overestimation at low values and underestimation at high values observed in the RF and SVM models. Additionally, the 95% confidence intervals depicted in **Figure 7C** illustrate the uncertainty of the CNN predictions, with relatively narrower intervals than those of RF and SVM, reflecting enhanced prediction stability.

#### 3.3.3 Prediction of nitrogen content in apple leaves based on PSO-CNN

As shown in **Figure 8B**, the PSO-CNN model demonstrates rapid and stable convergence during training. The *RMSE* decreases sharply in the initial stage and then gradually stabilizes, reaching a low value of approximately 0.05 with minimal fluctuations. This behavior indicates effective optimization of network hyperparameters and stable model convergence.

**Figure 9:**
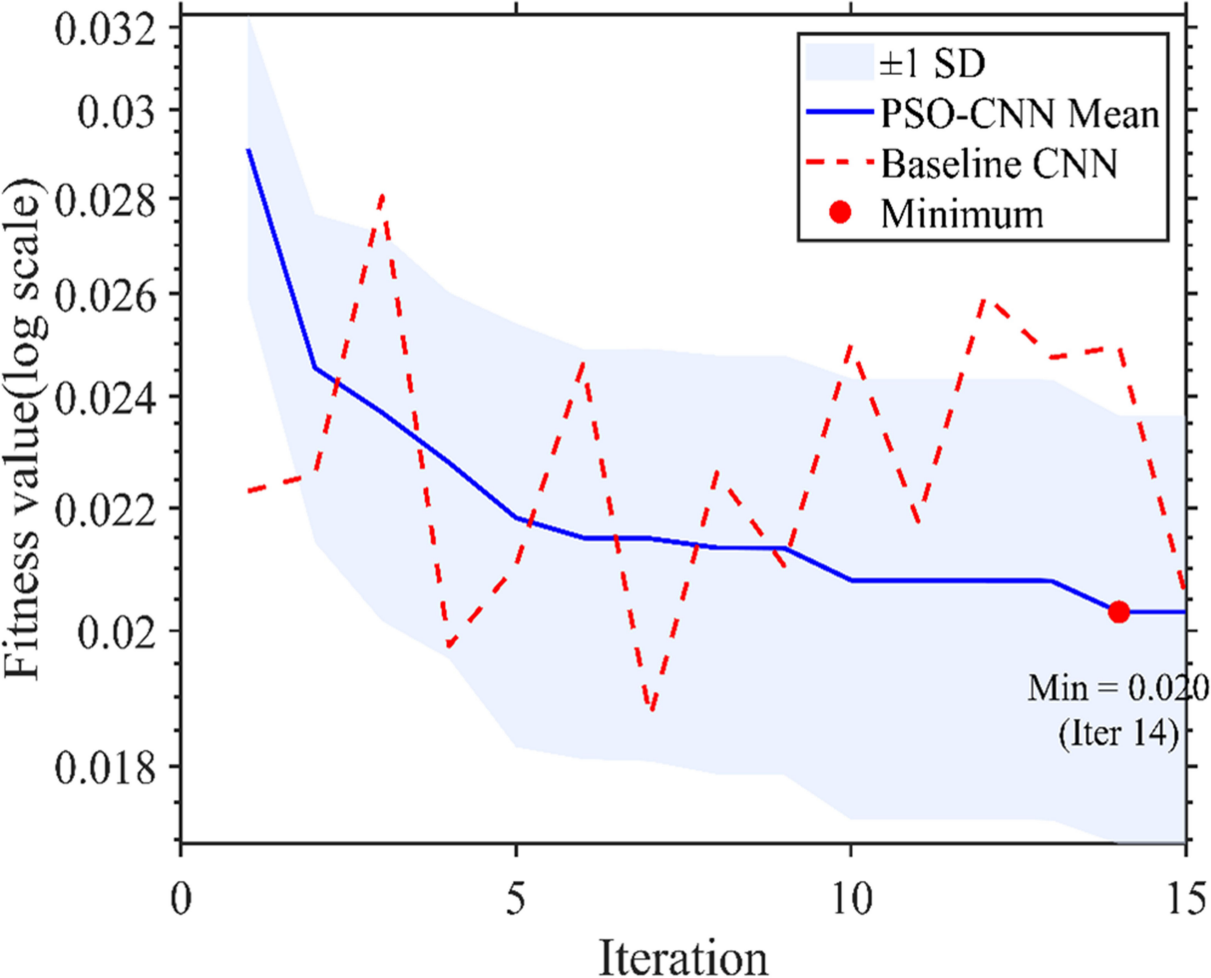
Optimization process of the PSO-CNN model over iterations. The blue solid line shows the mean fitness value, and the shaded region represents ±1 standard deviation (SD) based on n = 10 independent runs. The red dashed line indicates the fitness values of the baseline CNN model. The red marker denotes the minimum fitness value (0.020 at iteration 14). Fitness values are plotted on a logarithmic scale, with lower values corresponding to better model performance.

The convergence behavior of the PSO process is illustrated in **Figure 9**. The mean fitness value decreases rapidly during the initial iterations and stabilizes after approximately the 10th iteration. The shaded region (± 1 standard deviation) indicates relatively low variability across runs, reflecting stable optimization performance. The minimum fitness value (0.020) is achieved at the 14th iteration, suggesting that the PSO algorithm efficiently identifies near-optimal hyperparameter combinations within a limited number of iterations. In contrast, the baseline CNN exhibits larger fluctuations and generally higher fitness values. These results demonstrate that PSO enhances both convergence efficiency and optimization stability, showing greater robustness against data noise and outliers (Lawrence et al., 2021).

The prediction results of the PSO-CNN model are presented in **Figure 7D**. The model achieved *R²* values of 0.886 and 0.774 for the training and test sets, respectively, with corresponding *RPD* values exceeding 2.0, indicating excellent predictive performance. The scatter distribution aligns more closely with the 1:1 line, suggesting reduced systematic bias compared to the RF, SVM, and CNN models. Additionally, the 95% confidence intervals in **Figure 7D** are narrower and more consistent across the range of nitrogen content, indicating reduced prediction uncertainty and enhanced model robustness.

#### 3.3.4 Comparison and analysis of prediction model accuracy and model optimization

Overall, deep learning models outperformed traditional machine learning approaches, with the CNN achieving higher prediction accuracy than both RF and SVM. Further improvements were observed after PSO optimization, with the PSO-CNN model demonstrating the best overall performance among all models. Model comparisons indicate a consistent trend of improvement from SVM to RF, then CNN, and finally PSO-CNN. Compared to the CNN model, PSO-CNN increased the *R²* values for both the training and test sets by approximately 0.1. Additionally, the *RPD* improved by 0.765 for the training set and 0.409 for the test set, while the *RMSE* decreased by 0.033 and 0.024, respectively. These results clearly demonstrate enhanced prediction accuracy following PSO optimization. The performance gains can be attributed to the global optimization capability of PSO, which enables more effective hyperparameter tuning and improves the model’s ability to capture complex nonlinear relationships between spectral features and leaf nitrogen content.

#### 3.3.5 PSO-CNN inversion model verification

Model generalization and stability were evaluated on an independent dataset collected in Qixia City, including samples from different years, phenological stages, and orchard locations. Previous studies have demonstrated that leaf nitrogen content in apple trees varies significantly across growth stages (Li et al., 2025). Among these stages, the new shoot stage (NSS) and autumn shoot stage (ASS) are critical periods for nitrogen accumulation and redistribution, serving as representative indicators of leaf nitrogen status. Accordingly, four models were developed using 125 NSS samples collected in June 2023, while 118 ASS samples from September 2024 were used as an independent validation dataset to assess the generalization performance of the optimal PSO-CNN model.

**Figure 10:**
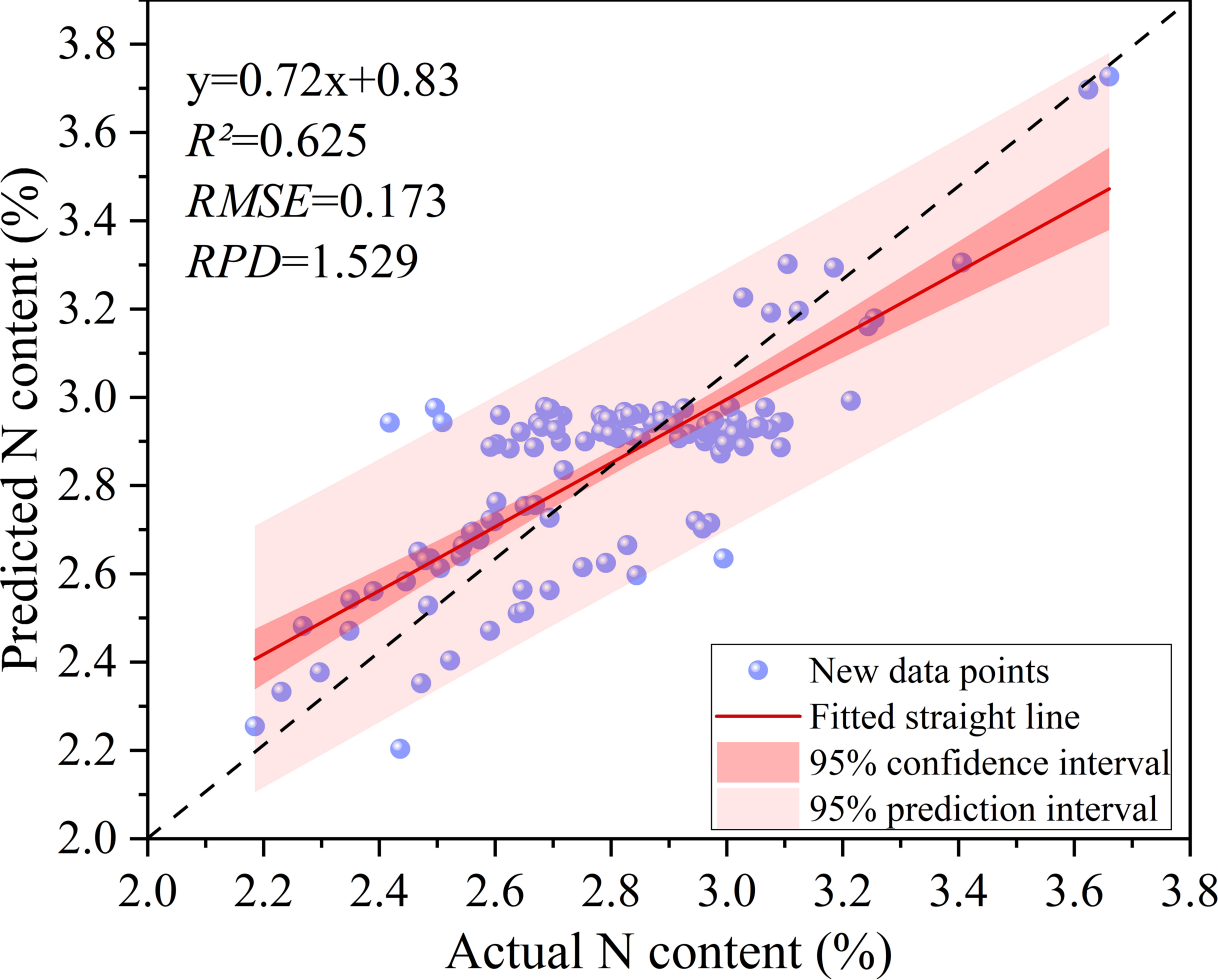
Prediction fit results for the new dataset (from the autumn shoot stop growing stage in 2024, and the sample size is n = 118).

Prediction performance on the independent dataset is presented in **Figure 10**. The coefficient of determination (*R²* = 0.625) indicates that the model explains 62.5% of the variance, reflecting moderate predictive capability. The regression slope (0.757) deviates from the ideal 1:1 line, suggesting a tendency to underestimate higher values. The *RMSE* (0.173) demonstrates acceptable prediction accuracy, and an *RPD* value greater than 1.5 indicates good model applicability for leaf nitrogen estimation across phenological stages. In addition, most observations fall within the 95% prediction interval, indicating that prediction errors remain within a reasonable range. The mean predicted values are stable and lie within the 95% confidence interval.

**Figure 11:**
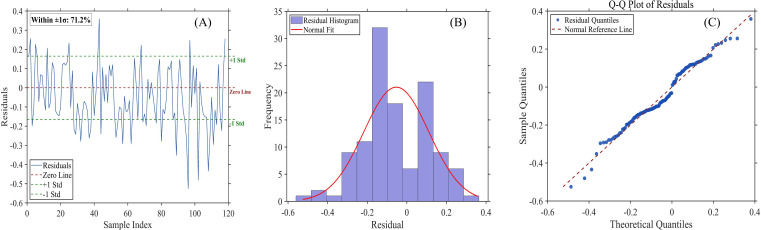
Residual analysis of the model. **(A)** Residuals plotted against sample index, with the zero-residual reference line (solid) and ±1 SD, standard deviation bounds (dashed). The label “Within ±1 SD: 71.2%” denotes the proportion of residuals that lie within one standard deviation of the mean. **(B)** Histogram of residuals with a fitted normal distribution curve. **(C)** Quantile–quantile (Q–Q) plot comparing sample residuals with theoretical normal quantiles. The residuals are approximately normally distributed, with the majority falling within ±1 SD. The sample size is n = 118.

Residual diagnostics further confirm the model’s robustness (**Figure 11**). The residual sequence (**Figure 11A**) shows no evident clustering or systematic trends, with 71.2% of residuals falling within ±1 standard deviation, which is close to the expected proportion under a normal distribution. The residual histogram (**Figure 11B**) is approximately symmetric around zero, indicating the absence of significant systematic bias. The Q–Q plot (**Figure 11C**) shows that most residuals closely align with the theoretical normal distribution line, suggesting approximate normality. Overall, these results demonstrate that the PSO-CNN model maintains stable and reliable predictive performance when applied to independent datasets across different phenological stages.

#### 3.3.6 Interpretability analysis of PSO-CNN input features based on SHAP

The black-box nature of deep learning models limits insight into their decision-making processes (Lu et al., 2025). Although model accuracy has been widely emphasized, interpretability remains underexplored. SHAP provides a transparent framework for quantifying the contributions of input variables to model predictions, thereby enhancing the interpretability and reliability of nitrogen estimation (Zhang et al., 2025).

**Figure 12:**
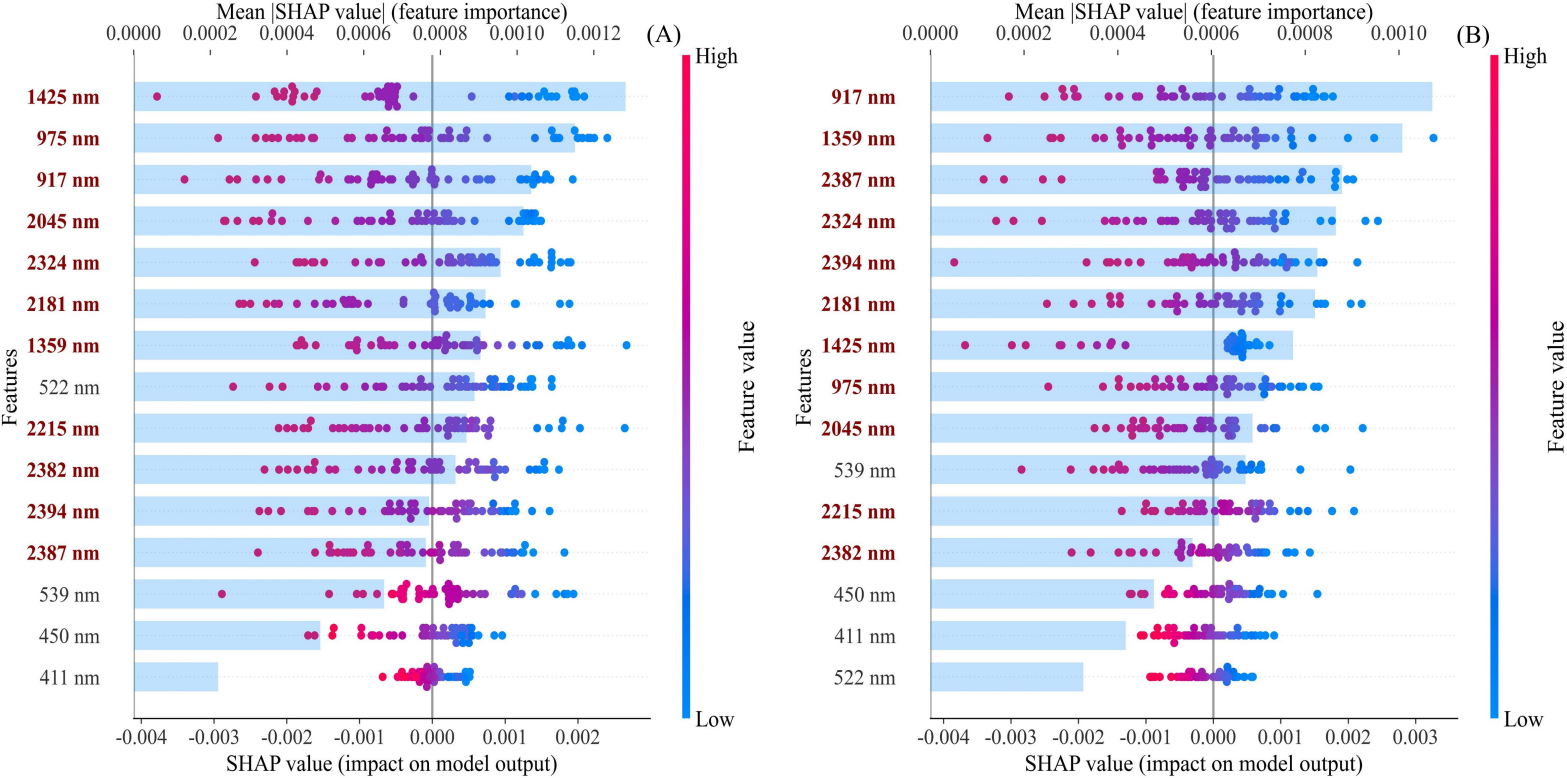
SHAP (Shapley Additive Explanations) beeswarm plots illustrating feature importance. **(A)** New shoot stop growing stage (NSS) in 2023 (n=125); and **(B)** Autumn shoot stop growing stage (ASS) in 2024 (n=118). Each dot represents an individual sample, with the x-axis indicating the SHAP value (contribution of each feature to the model output). Color represents the feature value, ranging from low (blue) to high (red). Features are ranked according to their mean absolute SHAP values. The wavelengths shown in red denote the near-infrared (NIR) and shortwave infrared (SWIR) regions.

SHAP analysis was applied to the optimal PSO-CNN model to characterize the contributions of spectral features to leaf nitrogen prediction. As shown in **Figure 12A**, the most influential spectral bands are at 1425 nm, 975 nm, 917 nm, and 2045 nm. These bands are primarily located in the near-infrared (NIR) and short-wave infrared (SWIR) regions, indicating that the model’s predictions mainly rely on spectral information related to leaf water status, mesophyll structure, and protein absorption during the NSS period. The SHAP value distribution reveals that higher reflectance values (red) are generally associated with negative SHAP values, whereas lower reflectance values (blue) are associated with positive SHAP values. This pattern suggests that reduced reflectance in these bands is linked to higher predicted nitrogen content, particularly in the SWIR regions (*e.g*., 1425 nm and 2045 nm), underscoring the importance of water- and protein-related absorption features.

For the ASS dataset (**Figure 12B**), the dominant features shift to wavelengths of 917 nm, 1359 nm, 2324 nm, and 2387 nm, all concentrated in the NIR and SWIR regions. The 917 nm band is sensitive to chlorophyll and leaf structure, while the 1359 nm, 2324 nm, and 2387 nm bands are associated with absorption by water and nitrogen-containing organic compounds, including proteins. A consistent SHAP pattern is observed, where lower reflectance values contribute positively to nitrogen prediction, whereas higher reflectance values have negative effects. This indicates that nitrogen estimation during the ASS stage is primarily driven by spectral responses related to leaf water content and protein status. These findings align with previous reports emphasizing the influence of water content and protein composition on leaf nitrogen estimation (Rahul et al., 2021).

Comparative analysis across phenological stages reveals that, despite variations in feature ranking, the model consistently prioritizes the NIR and SWIR bands. Specifically, the spectral regions around 917 nm, 1359–1425 nm, and 2045–2394 nm are highly important in both datasets. This consistency indicates stable spectral sensitivity across growth stages, with nitrogen estimation primarily driven by spectral features associated with leaf moisture and nitrogen-containing compounds. Such spectral dependence underscores the robustness of the PSO-CNN model under varying phenological conditions.

## 4 Discussion

### 4.1 Correlation between feature wavelength and leaf nitrogen content

The 15 characteristic wavelengths selected by the hybrid CARS–SPA algorithm had low RMSE values and were of physiological and optical relevance to the leaf nitrogen status. Reflectance in the visible region is strongly associated with photosynthetic pigments, whose synthesis depends directly on nitrogen availability. As a critical component of chlorophyll and carotenoids, nitrogen influences the pigment concentration and hence the spectral responses.

The bands at 411 nm and 450 nm are within the strong light-absorption regions of chlorophyll and carotenoids. Under nitrogen-sufficient conditions, an increase in pigment synthesis enhances blue-light absorption, resulting in lower reflectance. In contrast, nitrogen deficiency reduces the pigment content and leads to higher reflectance. The bands in the green region (522 nm and 539 nm) capture variation in the reflectance peak associated with chlorophyll concentration, consistent with previous findings (Hou et al., 2024). Unlike the strong absorption in the blue region, the magnitude and slope of the peaks in the green region are particularly sensitive to subtle changes in chlorophyll content. Higher nitrogen availability, which is associated with elevated chlorophyll concentrations, increases the relative absorption in the green region and reduces reflectance intensity.

Near-infrared bands (*e.g*., 917 nm and 975 nm) are predominantly influenced by the leaf internal structure and water content, and indirectly reflect the nitrogen-related physiological status. In the short-wave infrared (SWIR) region, spectral signals are dominated by molecular vibrational absorption. Because proteins represent the primary nitrogen pool in leaves, protein-related absorption features may be proxies for nitrogen content. For example, Peanusaha et al. (2024) incorporated protein content into a physical model for nitrogen estimation in grapevine leaves. Specifically, bands at 2045 nm, 2181 nm, and 2215 nm are associated with amide bonds and C–H vibrations in proteins. A higher protein content leads to stronger absorption and lower reflectance in these bands (Guo et al., 2019). In contrast, bands such as 2324 nm, 2382 nm, 2387 nm, and 2394 nm are associated with C–H vibrations in structural carbohydrates, including cellulose and lignin. Under nitrogen deficiency, carbohydrate accumulation may increase, thereby enhancing the reflectance signals in these bands. Thus, the interaction between carbon and nitrogen metabolism allows indirect assessment of the nitrogen status from spectral responses.

The selected characteristic wavelengths capture complementary information on pigment composition, leaf structure, water status, and biochemical constituents. This spectral sensitivity enables the deep learning model to effectively learn the relationship between reflectance and leaf nitrogen content, thereby improving the predictive performance of the model.

### 4.2 Model complexity evolution from traditional machine learning to PSO-CNN

Modeling approaches for crop nitrogen estimation differ in structural complexity and representation capacity. Conventional methods, such as RF and SVM, rely on shallow architectures and manually extracted spectral features, limiting their ability to capture complex nonlinear relationships. Although robust with small datasets, their feature representational capacity is limited.

A CNN model overcomes this limitation by employing hierarchical feature extraction, enabling automatic learning of nonlinear spectral representations. The effectiveness of CNN models for nitrogen estimation has been widely demonstrated (Pullanagari et al., 2021; Pourdarbani et al., 2021). However, CNN performance is highly sensitive to hyperparameter selection, and suboptimal settings with high-dimensional, small-sample datasets can lead to premature convergence in local optima or convergence instability (Hu et al., 2024).

To improve the training robustness, PSO replaced empirical tuning for global hyperparameter optimization (Li et al., 2021). The network architecture was unchanged, ensuring that the structural complexity was not increased. Critical parameters (*i.e*., learning rate, batch size, and regularization coefficient) were optimized using a fitness function that balanced validation error and model complexity. This strategy improved the parameter search efficiency and reduced premature convergence, consistent with previous findings (Wang et al., 2023).

The optimized model achieved a lower learning rate (0.0005), larger batch size (512), and stronger regularization (0.1), resulting in improved stability and reduced overfitting without increasing the computational cost. Compared with the baseline CNN, the PSO-CNN model attained superior performance (training: *R*² = 0.886, *RMSE* = 0.079, *RPD* = 2.579; test: *R*² = 0.774, *RMSE* = 0.095, *RPD* = 2.086). Overall, the performance gains arose from improved hyperparameter optimization rather than increased model complexity, enabling the PSO-CNN model to achieve a favorable balance between accuracy and efficiency for leaf nitrogen estimation.

### 4.3 Validation and interpretability of PSO-CNN model based on different phenological periods

The model was trained using leaf samples collected at the NSS stage and was evaluated on an independent ASS dataset, thereby providing a stringent test of cross-phenological transferability. Apple leaves undergo substantial changes in nitrogen allocation, water status, and biochemical composition across growth stages (Li et al., 2025), resulting in systematic variation in spectral reflectance, particularly in the NIR and SWIR regions. At the NSS stage, the leaves are characterized by a high chlorophyll content, active nitrogen assimilation, and high water content, together with a relatively loosely packed mesophyll structure that enhances internal light scattering (Delalieux et al., 2009). In the ASS stage, nitrogen is progressively redistributed to the fruit and perennial organs (Mahmoud and Hatem, 2021), accompanied by declines in the chlorophyll and water contents and increases in accumulation of structural compounds. These changes alter the spectral absorption and light-scattering properties, potentially weakening the spectral–biochemical relationships learned during model training. This shift partly explains the lower *RPD* (1.529) in cross-phenological validation. Nevertheless, the model showed acceptable predictive performance, indicating reasonable robustness across growth stages. Incorporation of multi-year and multi-phenological datasets may further improve the model generalization.

SHAP analysis provided additional insights into the phenological changes. For the NSS-trained model, bands associated with water status and protein absorption (*e.g*., 1425 nm, 975 nm, and 2045 nm) were major contributors, consistent with the elevated nitrogen accumulation and water content at this stage. In contrast, the ASS samples show increased importance of bands such as 917 nm, 1359 nm, and 2387 nm, which reflect changes in chlorophyll content, leaf anatomy, and accumulation of nitrogen-containing compounds. Despite the shifts in feature ranking, the model consistently prioritized NIR and SWIR regions associated with water status and protein content. These results indicate that the PSO-CNN model captures the underlying physiological mechanisms rather than stage-specific spectral patterns, enabling stable estimation of the nitrogen status across phenological stages.

### 4.4 Limitations and prospects

Despite its promising performance, the PSO-CNN model has several limitations. The relatively small sample size, constrained by the field sampling conditions, may limit model generalization and prediction stability. In addition, the PSO algorithm was implemented with a limited particle population and number of iterations to balance the computational cost and data availability. Although this configuration improves the model efficiency and reduces the risk of overfitting under a small sample size, it may restrict exploration of the hyperparameter space and increase the likelihood of suboptimal convergence.

Future work should extend the spatial and temporal sampling by incorporating additional orchards, growing seasons, and environments. Larger, more diverse datasets would support more extensive PSO optimization, enabling improved exploration of model configurations and enhancing robustness. The current analysis was limited to a single apple cultivar, which likely constrains the model transferability. Inclusion of multiple cultivars that differ in growth characteristics and nitrogen use efficiencies will be crucial for broader applicability of the model.

Although the PSO-CNN model effectively represents the relationship between hyperspectral features and leaf nitrogen content across phenological stages of apple, further research is required for its practical application. For example, the integration of nitrogen estimation with agronomic decision-making would support precision fertilization and facilitate the operational use of hyperspectral and machine learning approaches in orchard management.

## 5 Conclusion

Red Fuji apple leaves collected at the NSS and ASS growth stages in Qixia City (Shandong Province) were used to develop models for nitrogen estimation from spectral reflectance data. Following SD preprocessing and selection of spectral features using the CARS–SPA method, RF, SVM, CNN, and PSO-CNN models were constructed using 125 samples. Model generalization was evaluated using independent datasets for different orchards, years, and phenological stages. In addition, a SHAP-based interpretability analysis was conducted to clarify the spectral–physiological mechanisms underlying nitrogen estimation.

The PSO-CNN model achieved the best performance, while the CNN model outperformed the RF and SVM models. The PSO-CNN model had *R*² values of 0.886 (training dataset) and 0.774 (test dataset), and *RMSE* of 0.095 and *RPD* of 2.086 for the test dataset. Cross-stage validation demonstrated the acceptable predictive performance of the PSO-CNN model (*R*² = 0.625, *RMSE* = 0.173, *RPD* = 1.529), together with uniformly distributed residuals and no evident systematic bias, thus indicating satisfactory generalization and stability.

The superior performance of the PSO-CNN model was primarily attributable to PSO-based hyperparameter optimization rather than an increase in model complexity. In conjunction with SG–SD preprocessing and CARS–SPA-mediated feature selection, the PSO-CNN model effectively captured the nonlinear relationships between spectral reflectance and leaf nitrogen content. The model consistently prioritized NIR and SWIR bands associated with the leaf water status and nitrogen-containing compounds, thereby ensuring stable spectral sensitivity across two phenological stages. The proposed method offers a robust and accurate framework for estimating apple leaf nitrogen content, while improving the interpretability of deep learning models in fruit tree nutrient diagnostics.

## Data Availability

The raw data supporting the conclusions of this article will be made available by the authors, without undue reservation.
